# Parallel Evolution of Chordate *Cis-*Regulatory Code for Development

**DOI:** 10.1371/journal.pgen.1003904

**Published:** 2013-11-21

**Authors:** Laura Doglio, Debbie K. Goode, Maria C. Pelleri, Stefan Pauls, Flavia Frabetti, Sebastian M. Shimeld, Tanya Vavouri, Greg Elgar

**Affiliations:** 1 Systems Biology, MRC National Institute for Medical Research, Mill Hill, London, United Kingdom; 2 Dept. of Haematology, Cambridge Institute for Medical Research, Addenbrooke's Hospital, Cambridge, United Kingdom; 3 Center for Research in Molecular Genetics Fondazione CARISBO, Department of Experimental, Diagnostic and Specialty Medicine, University of Bologna, Bologna, Italy; 4 Department of Zoology, University of Oxford, Oxford, United Kingdom; 5 Institute of Predictive and Personalized Medicine of Cancer (IMPPC), Badalona, Barcelona, Spain; University of California Berkeley, United States of America

## Abstract

Urochordates are the closest relatives of vertebrates and at the larval stage, possess a characteristic bilateral chordate body plan. In vertebrates, the genes that orchestrate embryonic patterning are in part regulated by highly conserved non-coding elements (CNEs), yet these elements have not been identified in urochordate genomes. Consequently the evolution of the *cis-*regulatory code for urochordate development remains largely uncharacterised. Here, we use genome-wide comparisons between *C. intestinalis* and *C. savignyi* to identify putative urochordate *cis-*regulatory sequences. *Ciona* conserved non-coding elements (ciCNEs) are associated with largely the same key regulatory genes as vertebrate CNEs. Furthermore, some of the tested ciCNEs are able to activate reporter gene expression in both zebrafish and *Ciona* embryos, in a pattern that at least partially overlaps that of the gene they associate with, despite the absence of sequence identity. We also show that the ability of a ciCNE to up-regulate gene expression in vertebrate embryos can in some cases be localised to short sub-sequences, suggesting that functional cross-talk may be defined by small regions of ancestral regulatory logic, although functional sub-sequences may also be dispersed across the whole element. We conclude that the structure and organisation of *cis-*regulatory modules is very different between vertebrates and urochordates, reflecting their separate evolutionary histories. However, functional cross-talk still exists because the same repertoire of transcription factors has likely guided their parallel evolution, exploiting similar sets of binding sites but in different combinations.

## Author Summary

Vertebrates share many aspects of early development with our closest chordate ancestors, the tunicates. However, whilst the repertoire of genes that orchestrate development is essentially the same in the two lineages, the genomic code that regulates these genes appears to be very different, even though it is highly conserved within vertebrates themselves. Using comparative genomics, we have identified a parallel developmental code in tunicates and confirmed that this code, despite a lack of sequence conservation, associates with a similar repertoire of genes. However, the organisation of the code spatially is very different in the two lineages, strongly suggesting that most of it arose independently in vertebrates and tunicates, and in most cases lacking any direct sequence ancestry. We have assayed elements of the tunicate code, and found that at least some of them can regulate gene expression in zebrafish embryos. Our results suggest that regulatory code has arisen independently in different animal lineages but possesses some common functionality because its evolution has been driven by a similar cohort of developmental transcription factors. Our work helps illuminate how complex, stable gene regulatory networks evolve and become fixed within lineages.

## Introduction

Gene regulation is facilitated by the binding of transcription factors to specific sites in genomic DNA. Consequently, accurate control of gene expression in any cell is largely influenced by two variables; the presence of the transcription factor proteins themselves and accessibility to regulatory sites. During animal development, a highly complex and dynamic set of regulatory interactions must be precisely articulated in order to accurately direct the patterning of the embryo. This has resulted in the establishment of stable and robust, scale free gene regulatory networks (GRNs) [1], with high information content encoded into *cis-*regulatory modules (CRMs), where cohorts of transcription factors bind combinatorially to define a regulatory state [2], [3].

As a result of this, the largest and most highly conserved *cis-*regulatory sequences identified in vertebrate genomes are associated with transcription factor genes that regulate development [4], [5], reflecting both the complexity and precision required to co-ordinate common patterning mechanisms during embryogenesis. Furthermore, the vast majority of these conserved non-coding elements (CNEs) are not conserved at the sequence level in invertebrate genomes, where parallel sets of *cis-*regulatory sequences have evolved [6], [7]. Interestingly, a tiny handful of vertebrate CNEs do share some sequence similarity with amphioxus elements [8], a more distant [cephalo]chordate relative, and even with elements in protostomes [9]. Recently, a number of shorter regions of sequence homology (av. 45 bp at 55% identity) have been identified between *Ciona* and vertebrates, although they are not generally associated with orthologous genes in the two lineages, and a majority are transcribed [10].

Nevertheless, urochordates must exploit genomic sequence, in the form of CRMs, to orchestrate their own development, deploying a similar repertoire of genes to vertebrates and other animal lineages. Indeed, patterning of the early vertebrate embryo and *Ciona* larva bear a strong resemblance to each other, suggesting that the many aspects of urochordate development are very similar to that of vertebrates [11], even if the rate at which their genome sequence has evolved is relatively rapid compared with amphioxus [12]. Two important questions therefore are how, and when, did complex CRMs for embryonic patterning become established in the chordate lineage. Are similarities in urochrodate and vertebrate patterning orchestrated by long established CRMs pre-dating the divergence of the chordate lineages, or have entirely different genomic sequences been recruited and deployed as CRMs in urochordates and vertebrates?

In order to address these questions we have identified a large set of urochordate (*Ciona*) specific CNEs (ciCNEs) through comparison of the highly diverged *C. intestinalis* and *C. savignyi* genomes, and compare them with vertebrate CNEs. The evolutionary distance between the two *Ciona* genomes is considered to be greater than the distance between human and chick, providing a very low background of unconstrained conservation [12]. Support for this comes from a genomewide study of vertebrate and ciona species which showed that Ciona species evolve about 50% faster than vertebrates [13], with a genomewide average amino acid distance between *intestinalis* and *savignyi* of 0.3349 (compared with values of 0.3258 and 0.3735 for human∶chick and human∶frog respectively). Many of the ciCNEs are associated with developmental regulator genes; in some cases the same genes that harbour CNEs in vertebrates, despite an absence of identifiable sequence similarity between the CNEs themselves. We test a number of these ciCNEs using two independent transgenic reporter assays in zebrafish embryos, and find that a small number drive highly specific and reproducible patterns of reporter expression. We then examine the relationship between enhancer sequence and function by further dissecting these sequences. We also assay a number of ciCNEs in *C. intestinalis* embryos. Our findings suggest that despite a degree of regulatory cross-talk, there is little evidence to suggest that the majority of CNEs in urochordates and vertebrates share sequence ancestry. Although it remains possible that binding site reorganization and sequence drift have resulted in very diverged homologous vertebrate and urochordate sets of CNEs, an alternative simple explanation for our findings is that the two sets of CNEs have been recruited and hardwired into the genome independently, after their divergence from a common chordate ancestor, albeit shaped by a similar repertoire of transcription factors. Functional characterization of a larger set of chordate and vertebrate CNEs would likely prove useful in distinguishing between these two scenarios.

## Results

### Identification of a parallel set of ‘ciCNEs’ in urochordates

We compared the assembled genomes of *C. intestinalis* and *C. savignyi* to identify conserved non-coding DNA sequences (Methods). Our analysis is quite different from a previous whole genome comparative analysis performed on these two genomes to identify highly conserved non-coding sequences [14] in that we removed any sequences that overlapped with known transcripts or non-coding RNAs. Consequently our dataset of 2,336 sequences (Dataset S1) represents predominantly *Ciona* conserved non-coding elements (ciCNEs).

The length distributions of both *C. intestinalis* and human CNEs are skewed to the right, with a few very long CNEs in both sets (Figure S1). ciCNEs are on average 181.6 bp long, ranging from 94 bp to 1,883 bp, with median 156 bp. For comparison, the lengths of the 1,373 human CNEs defined by alignment of the human and *Fugu* genomes [5] range from 93 bp to 737 bp, with a median of 177 bp. The distribution of ciCNEs is slightly more skewed than the vertebrate CNEs, reflecting a large set of short CNEs together with some extreme cases of very long CNEs. A majority of the extremely long ciCNEs overlap predicted exons (data not shown). Therefore, we expect that the extremely long ciCNEs are most likely to be at least partly un-annotated coding sequences. By comparing the sequence conservation between the two sets of CNEs, we find that ciCNEs are also less conserved than vertebrate CNEs. ciCNEs range from 71.0% to 96.8% sequence identity, with a median of 81.7%, while vertebrate CNEs range from 67.8% to 97.9%, with a median of 84.6% (based on human-*Fugu* pairwise comparisons). Therefore, ciCNEs are both shorter and less conserved than vertebrate CNEs, possibly reflecting a lower sequence constraint, or a simpler regulatory module structure in urochordates than in vertebrates.

We then tested whether CNEs cluster near developmental regulatory genes in the *C. intestinalis* genome as they do in vertebrate and nematode genomes. By assigning 2,146 ciCNEs to their closest protein coding genes (190 ciCNEs are on unplaced contigs containing no genes), we identified 1,289 ciCNE-associated genes (on average 1.7 ciCNEs per gene). Using the same approach, 1,373 human CNEs [5] are assigned to 397 CNE genes (on average 3.5 CNEs per gene). For this genome-wide comparison of human and *Ciona* CNEs we used proximity to assign genes to CNEs, however we expect that the numbers of CNE-associated genes are over-estimated as it is known that enhancers (and CNEs) can lie far from their targets. The number of CNE-associated genes in *Ciona* is likely to be exacerbated by the fact that the *Ciona* genome is highly fragmented. Nevertheless, in common with vertebrate CNEs, we found that ciCNE-genes are enriched for homeodomain-like (log-odds ratio = 2.03, p-value<2.2e-16), winged helix repressor (log-odds ratio = 1.71, p-value = 6.72e-11), HMG1/2 (log-odds ratio = 1.49, p-value = 1.43e-3) and zing finger C2H2 (log-odds ratio = 0.71, p-value = 1.52e-3) domains. In addition, we also found enrichment for several signalling domains that we previously saw overrepresented among nematode but not vertebrate CNE-associated genes. These domains include EGF-like (log-odds ratio = 0.68, p-value = 2.46e-3), laminin G (log-odds ratio = 1.89, p-value = 1.70e-4), cadherin (log-odds ratio = 1.71, p-value = 4.35e-4) and pleckstrin-like (log-odds ratio = 0.95, p-value = 7.92e-4). Using a compiled set of transcription factors and signalling genes in the *C. intestinalis* genome [15], we found that both types of genes are highly enriched in the ciCNE-associated gene set (log-odds ratio = 1.78, p-value<2.2e-16 and log-odds ratio = 1.43, p-value = 1.60e-10, respectively). Therefore, in terms of the protein domains they encode, the types of genes associated with CNEs in the *C. intestinalis* genome are consistent with the genes associated with CNEs in both non-chordate invertebrates [7] and vertebrates [5], perhaps reflecting the evolutionary position of *C. intestinalis* as an invertebrate chordate.

We then looked to see if the same genes are associated with CNEs in both urochordates and vertebrates. Among the ciCNEs-associated genes there are 32 Ciona genes orthologous to 38 human genes also associated with CNEs (orthology was determined using EnsemblCompara [16]) (Table S1). Interestingly, several of the *C. intestinalis* genes associated with multiple CNEs are orthologous to human genes also associated with multiple CNEs. For example, human *PTCH1* is associated with 3 CNEs and its *C. intestinalis* orthologue is associated with 4 ciCNEs. We note that most (21/32) ciona genes have multiple orthologues in human. So for example, two paralogous human genes, *MEIS1* and *MEIS2*, are associated with 10 and 42 CNEs respectively whilst their *C. intestinalis* orthologue is associated with 10 ciCNEs. The fact that orthologous genes in human and *Ciona* are associated with multiple CNEs further suggests that CNEs are associated with specific regulatory genes. Finally, we identified at least 45 ciCNEs that overlap with a limited number of functionally annotated cis-regulatory regions in the ANISEED database [17]. ANISEED is a database of genomic and functional information, such as gene expression patterns of genes, for ascidian genomes including those of *Ciona intestinalis* and *Ciona savignyi*.

**Figure 1:**
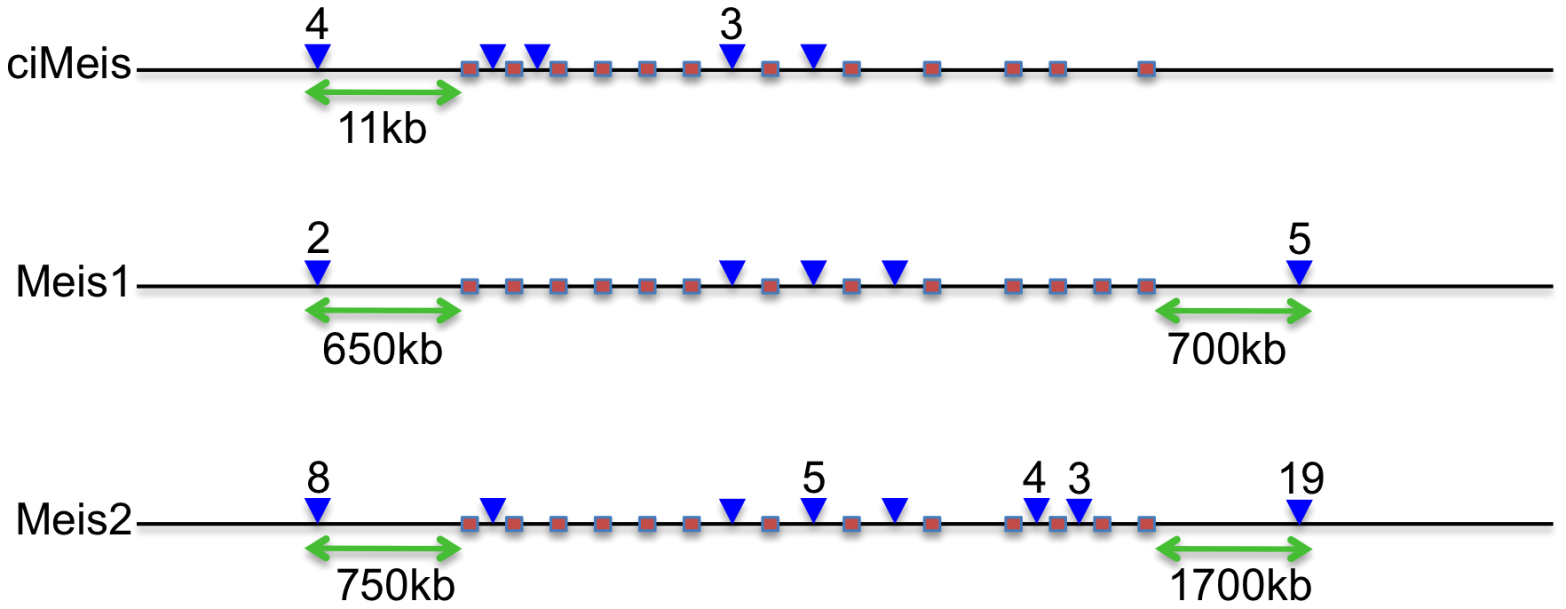
Relative positions of CNEs in *Ciona* and vertebrate Meis genes. Pink boxes denote coding exons showing similar gene structures in all 3 genes (*Ciona* lacks the twelfth exon). Blue arrowheads denote CNE positions (with numbers above if more than 1). Green arrows indicate the most distal upstream and downstream distances of CNEs from the coding sequence in each case. Not to scale.

It is intriguing that in many cases, CNEs are found next to the same gene in vertebrates and *Ciona* and yet they bear no observable sequence similarity to each other, despite being highly conserved within their respective lineages. Furthermore, their spatial organisation is very different. The *Ciona Meis* gene has 10 proximal CNEs of which 4 are upstream and the remaining 6 are dispersed across the first seven introns of the gene (Figure 1). This is in contrast with the distribution of CNEs around vertebrate *MEIS1* and *MEIS2*, where a majority of CNEs in each case are positioned in introns towards the end of the gene or downstream of the coding sequence. In the case of the human genes, the CNEs are often hundreds of kilobases from the coding sequence. This suggests that CNEs might have evolved independently in the two lineages but have then become fixed relatively early in their history, particularly in vertebrates. Nevertheless, the genes they co-associate with play very similar roles in each lineage and so we were interested to see if ciCNEs could function as spatio-temporally specific enhancers in zebrafish embryos, a model vertebrate for this type of study.

To select a subset of ciCNEs for experimental testing of enhancer activity, we first identified all ciCNEs that are associated with genes where the orthologous human gene is also associated with CNEs. We then narrowed down the list of candidate ciCNEs by considering only those associated with genes that have known and specific expression profiles during development according to the ANISEED database [17]. We also avoided the cases where the human CNE cluster is close to multiple candidate target genes and the cases where the predicted target gene (from Woolfe et al, [5]) is not the nearest gene to the CNE. From the remaining, we selected a subset of 22 candidate ciCNEs associated with nine different *Ciona* genes for experiments (Table 1; Text S1).

**Table 1:** Summary of GFP reporter expression driven by ciCNEs at 48*Tol2* and co-injection strategies in zebrafish embryos.

*Tol2* strategy							Co-injection strategy
Human gene name	*Ciona* Ensembl gene	# of *Ciona* CNEs	CNE Name	# of embryos (injections)	% embryo with phenotype	expression profile	
***PAX6***	ENSCING00000006503	2	Pax6_ciCNE1	256 (2)	Negative		
			**Pax6_ciCNE2**	**195 (3)**	**12**	**spinal cord sensory neurons and pre-otic ganglia neurons**	4/142 (neurons) (2.8%)
***OTX1/OTX2***	ENSCING00000008859	1	Otx_ciCNE1	150 (3)	Negative		
***NKX2-4***	ENSCING00000006231	3	Nkx_ciCNE1	146 (2)	4	sensory pre-otic ganglion	
			Nkx_ciCNE2	150 (2)	Negative		
			Nkx_ciCNE3	110 (2)	Negative		
***OTP***	ENSCING00000004120	1	Otp_ciCNE1	112 (2)	Negative		
***PHOX2B-like***	ENSCING00000009645	1	PhoxB_ciCNE1	120 (2)	Negative		
***MEIS1/MEIS2***	ENSCING00000005356	10	**Meis_ciCNE1**	**180 (2)**	**50**	**motor neurons and interneurons**	40/184 (neurons) (22%)
			Meis_ciCNE2	160 (2)	5	sensory post-otic ganglion	
			Meis_ciCNE3	116 (1)	Negative		
			Meis_ciCNE4	240 (3)	6	sensory post-otic ganglion	
			Meis_ciCNE5	160 (2)	Negative		
			Meis_ciCNE6	360 (3)	Negative		
			Meis_ciCNE7	168 (2)	Negative		
			Meis_ciCNE8	315 (3)	4	mixed population of sensory and interneurons	
			Meis_ciCNE9	236 (3)	Negative		
			**Meis_ciCNE10**	**285 (3)**	**20**	**spinal cord sensory neurons (Rohon-beard) and trigeminal ganglion neurons**	Less than 1% (neurons)
***EBF3***	ENSCING00000003209	1	Ebf3_ciCNE1	70 (1)	Negative		
***ZFHX1B***	ENSCING00000006930	2	Zfhx_ciCNE1	180 (2)	Negative		
			Zfhx_ciCNE2	No PCR	N/A		
*HHEX*	ENSCING00000001270	1	Hhex_ciCNE1	**321 (3)**	**12**	**macrophage-like**	20/82 (24%)

### Analysis of ciCNEs in zebrafish embryos

We independently tested 21 out of the 22 *Ciona* CNEs (one CNE failed to amplify during PCR), firstly exploiting a co-injection strategy using a minimal beta-globin promoter [5], and secondly through direct cloning into a *Tol2* vector with a c-fos promoter [18]. Whilst levels of GFP reporter expression were generally stronger using the *Tol2* vector, presumably due to more efficient integration and therefore reduced mosaicism, the results were highly reproducible between the two approaches. Four out of the 21 CNEs give robust and reproducible patterns of restricted GFP expression at either 24 or 48 hours post fertilisation (hpf) using both methods (Table 1). Two of these CNEs were from the *Meis* gene locus, one was from the *Pax6* region and the other resides within the only intron of the *Hhex* gene in both *Ciona* and vertebrates. A further four elements were able, in around 5% of embryos, to drive reporter expression in *Tol2* constructs only, but these were considered too weak to merit further analysis.

### A subset of *Ciona* CNEs drive highly specific and reproducible patterns of expression in zebrafish embryos using *Tol2* transgenesis

**Figure 2:**
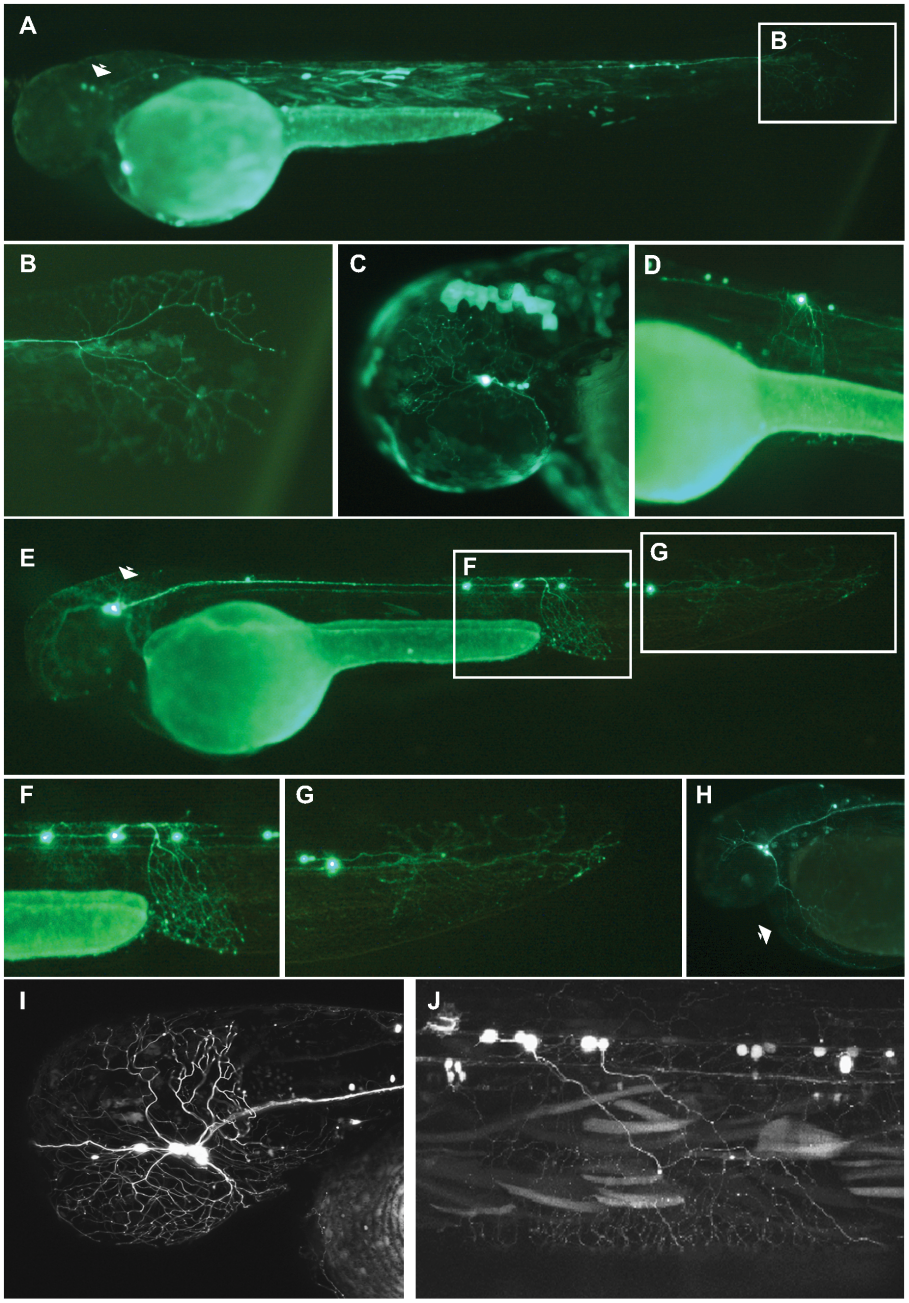
Analysis of Pax6_ciCNE2 and Meis_ciCNE10 in zebrafish embryos. Pax6_ciCNE2 drives GFP expression in both cranial ganglia (A, C) and spinal cord sensory neurons (B, D) at 48 hrs post fertilization. (B, D) High magnification view of GFP expression detectable in neuronal projections extending into the tail fin and along the spinal cord. (E) Meis_ciCNE10 drives GFP expression in the nervous system at 48 hpf. GFP is strongly expressed in the cell body and in the peripheral process of Rohon-Beard neurons innervating the ventral fin fold (F) and the tail fin (G). (H) GFP expression is detected in trigeminal ganglion neurons and in their process innervating the yolk sac (arrow). (I, J) Confocal analysis showing GFP expression in sensory neurons of the trigeminal ganglion innervating the head as well as in central axons innervating the hindbrain (I) and in projections of Rohon-Beard neurons (J).

We looked for consistent and reproducible patterns of GFP reporter expression in cell types other than muscle (we routinely see muscle expression in transient analyses with *Tol2*) in at least 10% of embryos screened for any particular ciCNE (Table 1). At 48 hpf, Pax6_ciCNE2 drives GFP expression in cranial ganglia and sensory neurons (Figure 2A, C) in 12% of screened embryos. More specifically, GFP is detectable in the sensory neurons innervating the tail fin (Figure 2B) and along the spinal cord (Figure 2D).

The two positive ciCNEs associated with the *Meis* gene drive very different patterns of GFP expression. Meis_ciCNE10 drives GFP expression in neuronal cells (Figure 2E) in 20% of embryos screened. At 48 hpf, GFP is readily detected in Rohon-Beard neurons (Figure 2F), including those innervating the tail fin (Figure 2G) as well as in trigeminal ganglion neurons (Figure 2H). GFP is observed in both cell bodies and axonal projections. A more detailed confocal analysis shows strong GFP fluorescence into the projections of the Rohon-Beard neurons and trigeminal ganglion, extending to the hindbrain (Figures 2I, J).

**Figure 3:**
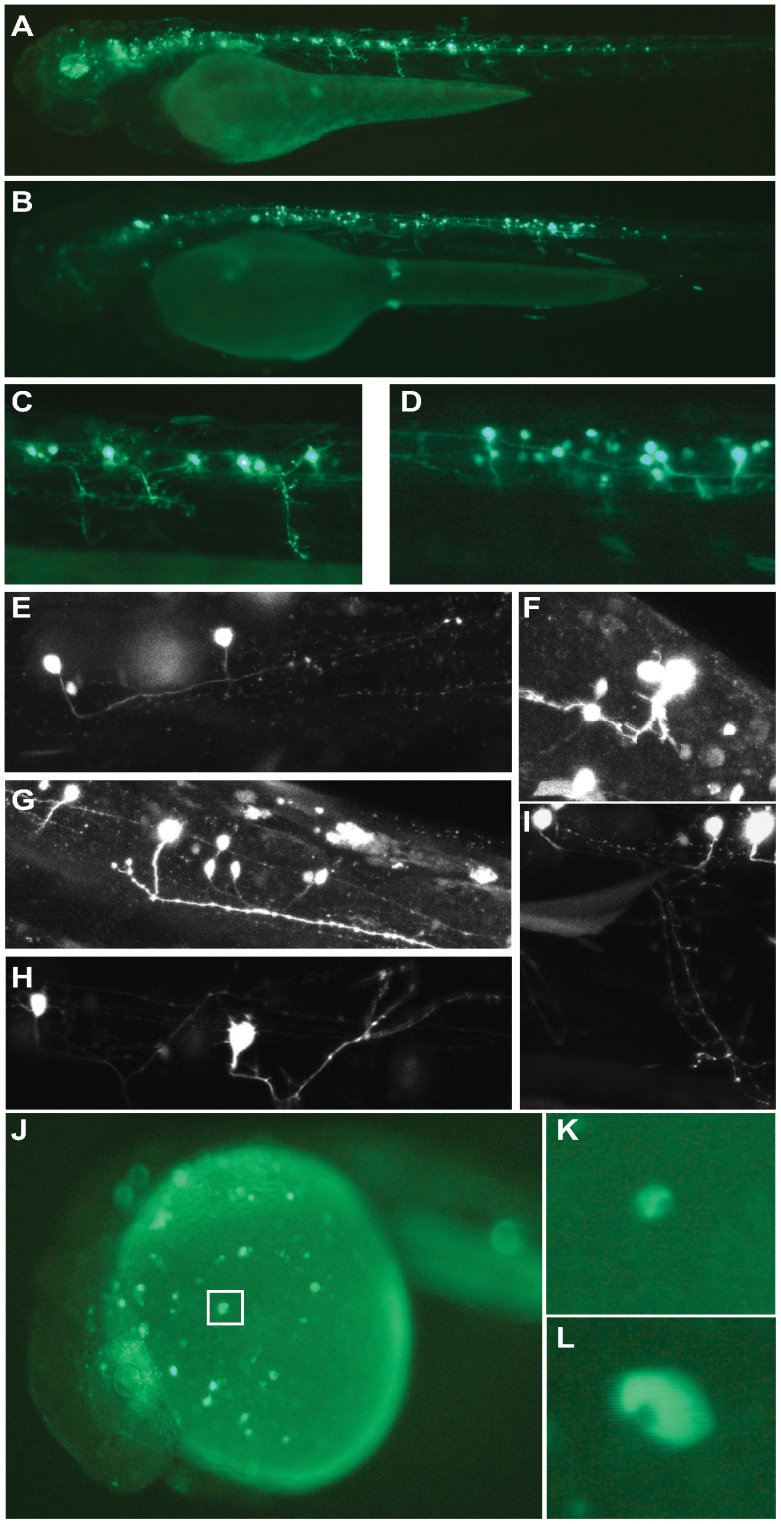
Analysis of Meis_ciCNE1 and Hhex_ciCNE1 in zebrafish embryos. Meis_ciCNE1 drives GFP expression in spinal cord motor neurons (A, C) and interneurons (B, D). Confocal analysis allowed to identify morphological subtypes of interneurons (E–G) and motor neurons (H, I). Intronic Hhex_CNE1 drives GFP expression in a discrete population of blood cell precursors, possibly macrophages (J–L).

Injection of Meis_ciCNE1 drives a very robust pattern of GFP expression (Figure 3A–I). Remarkably, in over 50% of embryos screened, GFP expression is detected in motor neurons (Figure 3A, C) and interneurons (Figure 3B, D). Confocal microscopy allowed us to identify morphological subtypes of interneurons and motor neurons. Two classes of descending interneurons (Figure 3E), one class of ascending interneurons (Figure 3F) and one class of bifurcating interneurons (Figure 3G) were GFP positive, as were as at least two subtypes of primary motor neurons (Figure 3H, I). It should be noted that meis1 has been identified as a gene potentially involved in interneuron migration [19].

Finally, in embryos injected with the Hhex_ciCNE1 GFP expression was detected in cells of the hematopoietic lineage (Figure 3J) The size and morphology of the cells resemble macrophages (Figure 3K, L). In zebrafish, a specific lineage of early macrophages differentiate in the yolk sac before the onset of blood circulation [20].

### De-lineation of the activating potential of ciCNEs in zebrafish embryos

Previously we have shown that evolutionarily conserved aspects of enhancer function often reside in core regions of a CNE sequence [21]. In order to examine whether sub-regions of ciCNEs are sufficient to drive GFP expression in zebrafish, we carried out an extensive functional analysis of sub-sequences of the pax6 ciCNE and the two meis ciCNEs.

**Figure 4:**
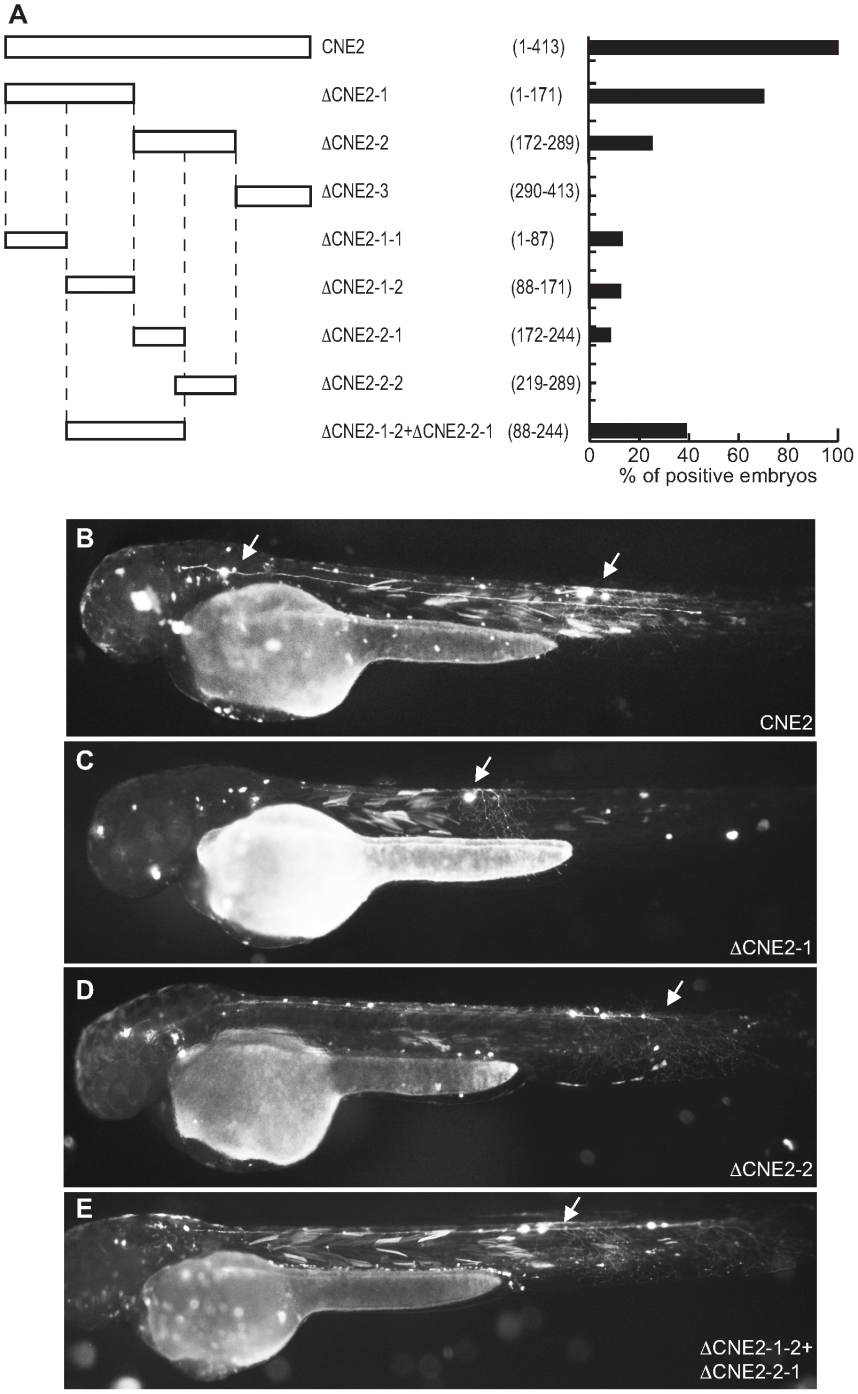
Functional analysis of Pax6_ciCNE2 deletion constructs. (A) Schematic representation and quantification of the constructs injected in the zebrafish embryos to examine their enhancer activity. The numbers in parentheses indicate the length of each construct. (B) View of a 48 hpf embryo injected with the full length CNE2 expressing GFP in sensory neurons (arrow). In the embryos injected with DCNE2-1 (C) or DCNE2-2 (D) GFP is detected in sensory neurons (arrow). The minimal construct DCNE2-1-2+DCNE2-2-1 (E) is able to drive GFP expression in neurons along the spinal cord (arrow).

**Figure 5:**
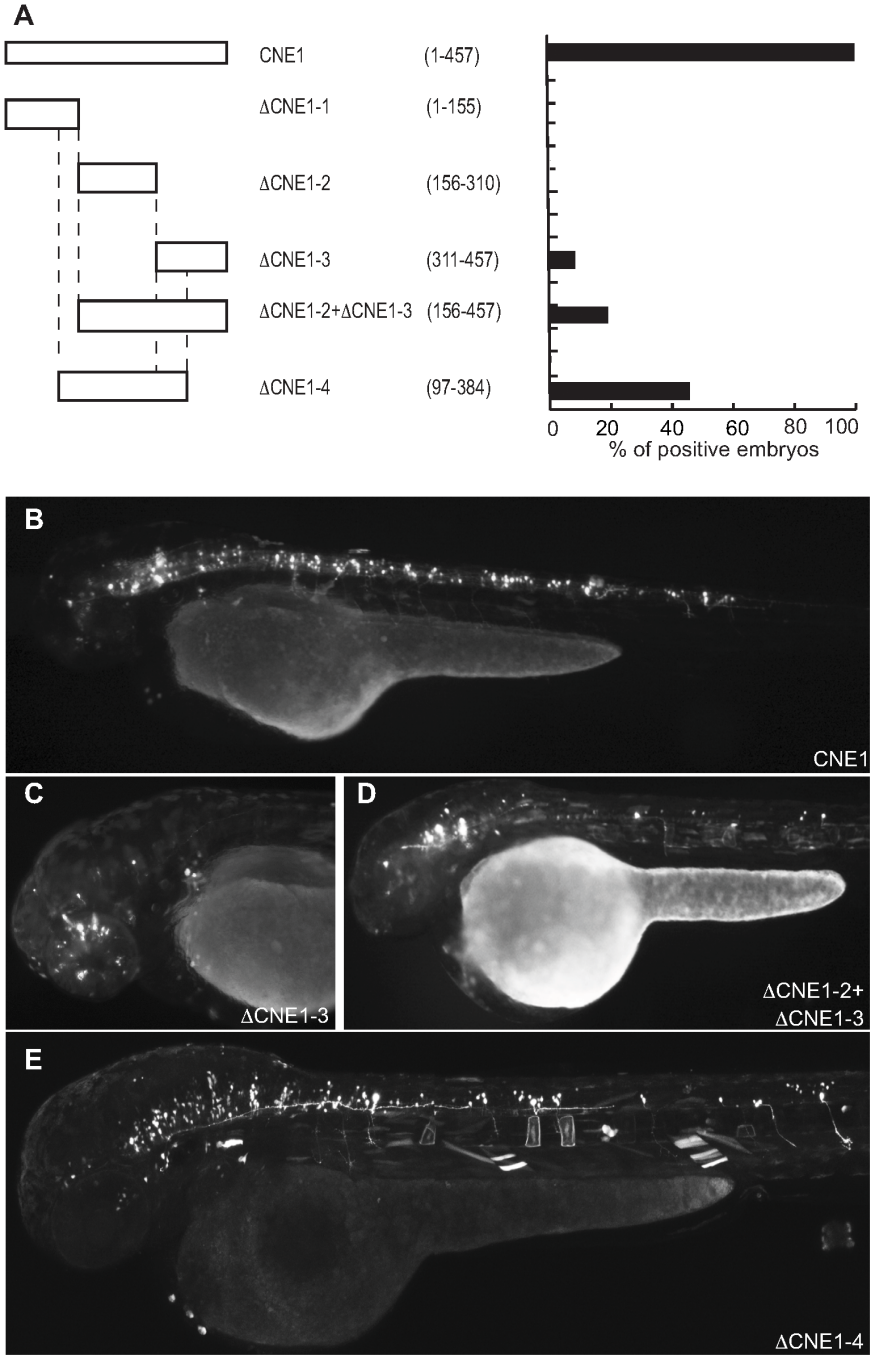
Deletion analysis of Meis_ciCNE1 construct. (A) Scheme and quantification of the deletion constructs injected into zebrafish embryos and analysed for GFP expression at 48 hpf. The numbers in parentheses indicate the length of each construct. (B) Embryo injected with the full length CNE1 show GFP expression in the brain and in spinal cord motor- and interneurons. (C) Injection of DCNE1-3 drives GFP expression only in a few neurons in the head. (D) In the embryos injected with DCNE1-2+DCNE1-3 construct few motor- and interneurons are detected along the spinal cord. (E) In embryos injected with the construct DCNE1-4 GFP expression shows a pattern similar to full length, labelling both spinal cord interneurons and motor neurons as well as cells in the brain.

Pax6_ciCNE2 is 413 nucleotides (nt) in length and was initially divided into three non-overlapping segments (Figure 4A) and the relative activity of each sub-region compared with the whole ciCNE (Figure 4B). Only the first two regions are able to activate GFP expression (Figure 4C, D), with the first 171 nt being most active. A further delineated region spanning nt 88–244 is able to drive the same patterns of GFP reporter expression as the entire ciCNE, but in a little under half the number of embryos (Figure 4E). Meis_ciCNE1, a particularly strong enhancer in zebrafish, is 457 nt in length and was similarly initially divided into three non-overlapping segments (Figure 5A). Only the most 3′ region shows any activity (Figure 5C) and this is both anteriorly restricted and observed in ten times fewer embryos than the full length element (Figure 5B). Whilst fusing the middle and 3′ regions together gave a small increase in the number of embryos driving GFP (Figure 5D), a larger central core region, encompassing nt 97–384, is able to drive more robust and comprehensive expression, re-capitulating the pattern driven by the full-length ciCNE (Figure 5E).

**Figure 6:**
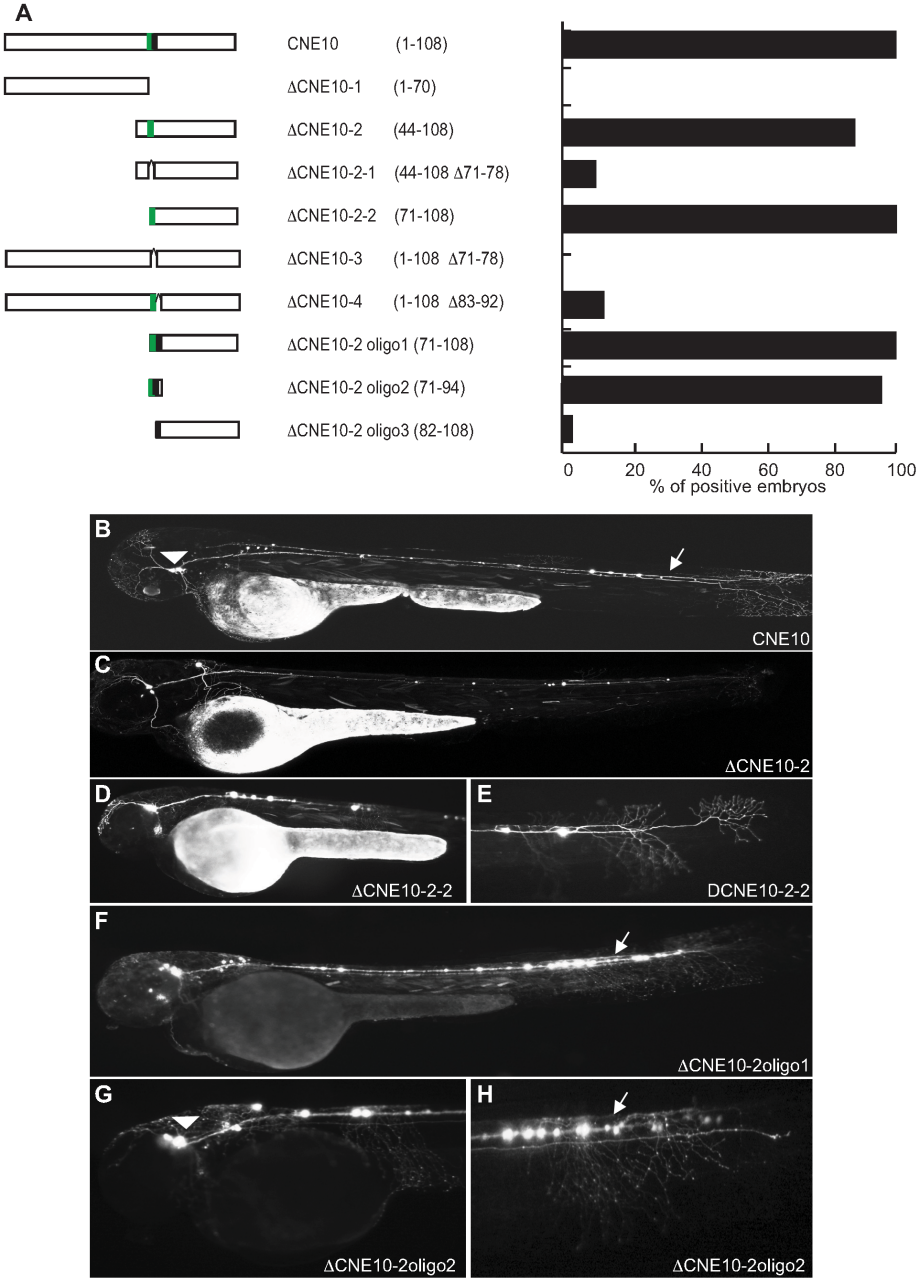
Deletion analysis of Meis_ciCNE10 construct. (A) Schematic view and quantification of the constructs injected in the embryos and analyzed at 48 hpf. The numbers in parentheses indicate the size of each construct. The green area represents a putative Pbx-Hox site (nt71–79) and the black area a random 10 bp sequence (nt 83–92). (B) View of an embryo injected with the full length CNE10 showing GFP expression in the trigeminal ganglion (arrowhead) and spinal cord sensory neurons (arrow). The two elements DCNE10-2 (C) and DCNE10-2-2 (D, E) retain the same enhancer potential as the full length construct (B). Embryo injected with the 37 bp DCNE10-2oligo1 (F) or with the 24 bp DCNE10-2oligo2 (G, H) show strong GFP expression in sensory neurons (arrow) and trigeminal ganglia (arrowhead).

Meis_ciCNE10 is a relatively short element, just 108 nt in length. Initially, this element was divided into two overlapping sub-regions ΔCNE10-1 and ΔCNE10-2 (Figure 6A), where all the detectable enhancer activity was confined to the second segment (Figure 6B, C). Further definition of the ciCNE resulted in a 3′ fragment encompassing nt 71–108 (ΔCNE10-2-2) which retains the same enhancer potential as the full element (Figure 6D, E). Deletion of a putative Pbx-Hox site at nt 71–79 from ΔCNE10-2 (ΔCNE10-2-1) or from the full length ciCNE (ΔCNE10-3) results in loss of enhancer potential. However, enhancer activity is also lost on deletion of nt 83–92 from the full length ciCNE (ΔCNE10-4). Further synthetic constructs were then made by annealing complementary oligonucleotides representing nt 71–108 (ΔCNE10-2 oligo1 (Figure 6F)), nt 71–94 (ΔCNE10-2 oligo2 (Figure 6G, H)) and nt 82–108 (ΔCNE10-2 oligo3) resulting in the delineation of a minimal sequence of just 24 nucleotides (nt 71–94, 5′ tgattaatatttcataatgcacta 3′) that is sufficient to re-capitulate both the strength and varied pattern of GFP expression of the full length element.

**Figure 7:**
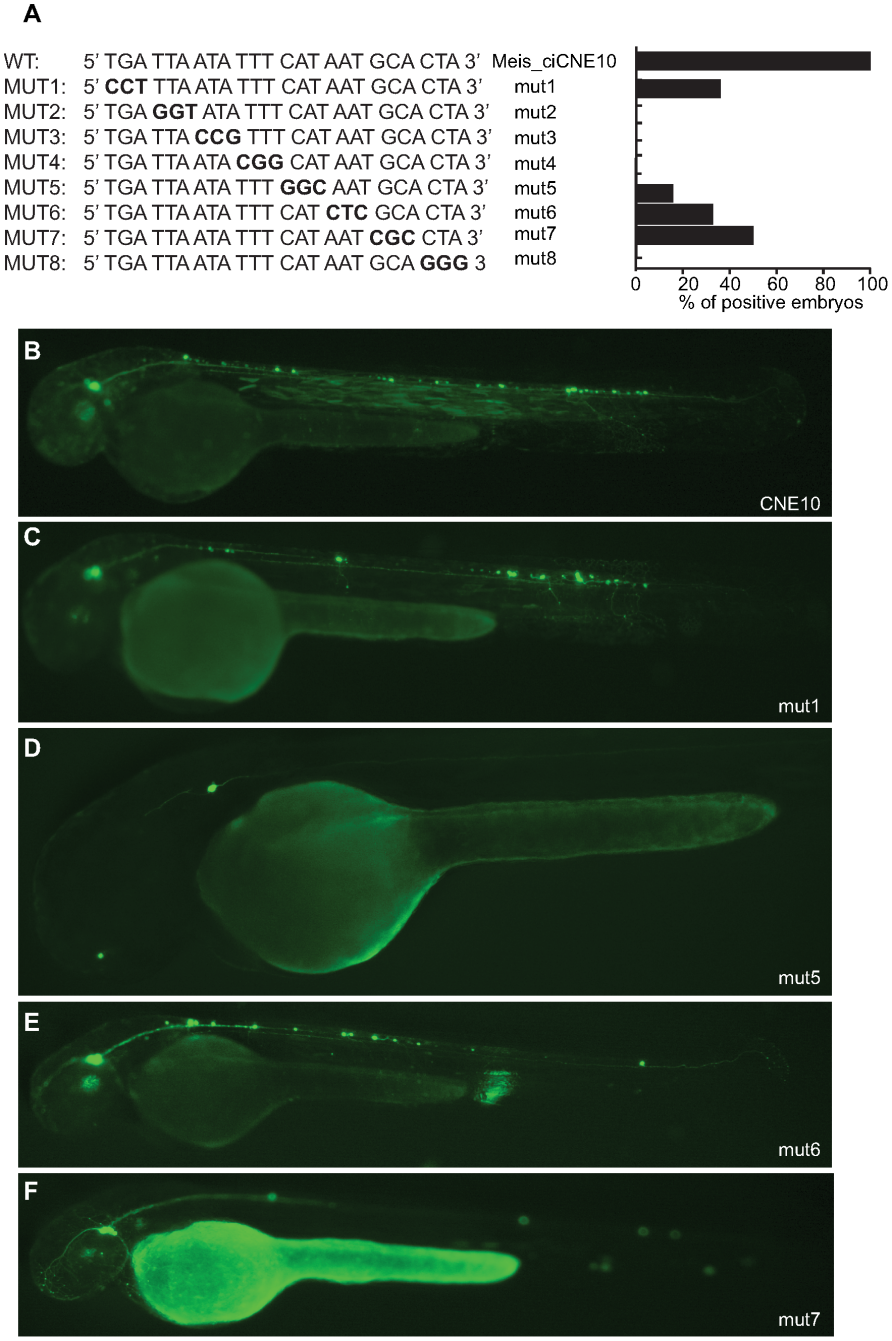
Mutational analysis of putative binding site in CNE10. (A) Schematic representation and quantification of mutations introduced in the 24 nt core sequence of CNE10. Mutated triplets are shown in bold. (B) Embryos injected with wt CNE10 show GFP expression in the nervous system at 48 hpf. (C, E) Embryos injected with mut1 and mut 6 show a GFP expression pattern similar to the wt construct, whereas injection of mut5 (D) drives GFP expression only in few neuronal cells. (F) In embryos injected with mut7 GFP expression is mainly detected in trigeminal ganglion neurons.

Trinucleotide site-directed mutagenesis across this region (Figure 7) identifies a critical 12 nucleotide motif, (5′ ttaatatttcat 3′) rich in A+T, and containing strong binding sites for helix-turn-helix homeodomain transcription factors, a diverse group of proteins that play important roles in developmental patterning. However, expression is considerably weaker at all mutated positions across the 24mer, suggesting that, as is generally the case, any homeodomain binding protein might be binding co-operatively alongside other factors across this site. Of particular note is the fact that the first 8 nucleotides of the 24mer sequence represent a perfect canonical Pbx/Hox site (tgatnnat), a bipartite site that itself forms a close relationship with meis proteins, and a motif that is strongly enriched in vertebrate CNEs [22].

We searched for sequence similarity to the 24 nt sequence (5′ tgattaatatttcataatgcacta 3′) that drives highly specific neuronal expression in zebrafish embryos and found no identical sequences in any of the organisms in Ensembl [23] except for the known match close to the *Meis* gene in *C. intestinalis*. We also profiled the 24mer for transcription factor binding sites in JASPAR [24] and TRANSFAC [25], predicting a large number of possible sites, including a 13 nt match to the binding site of the Oct domain binding transcription factor POU3F2, a protein known to be involved in neurogenesis in the central nervous system (CNS) [26].

The above experiments demonstrate that despite the absence of sequence conservation between ciona and vertebrate CNEs, 4 out of 21 ciona elements can act as enhancers in zebrafish. Extensive analysis of subsequences of these elements shows that in all cases the minimal functional CNE is at least 12 nt long. This suggests that these ciona elements are recognized and co-ordinately bound by more than one transcription factor in order for them to act as robust developmental enhancers in zebrafish.

### Analysis of ciCNE enhancer activity in *Ciona* embryos

We next assessed the activity of selected ciCNEs in *Ciona* embryos. We focused on well-annotated genes, particularly those with known expression patterns at the tailbud stage of development when major tissue types have been established and transgene assays are viable. Seventeen ciCNEs were assessed, three that had shown activity in zebrafish assays (Pax6_ciCNE2; Meis_ciCNE1; Meis_ciCNE10) and fourteen others (only Pax6_ciCNE1, Meis_ciCNE7, Zfhx_ciCNE1 and Hhex_ciCNE1 were not tested). These were cloned into the reporter vector pCES and electroporated into *Ciona* zygotes.

**Figure 8:**
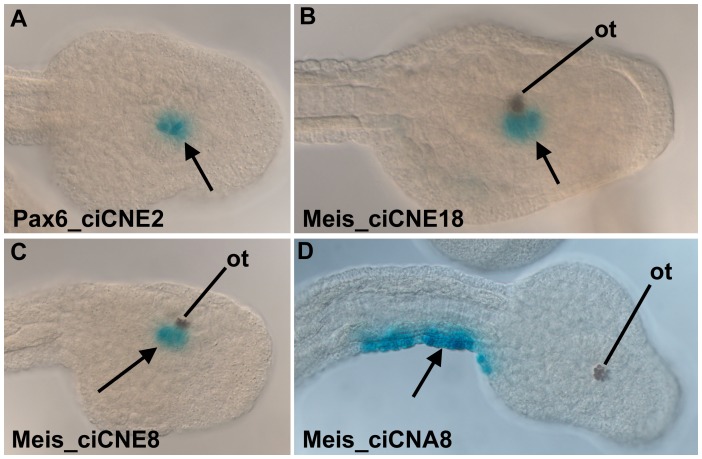
Analysis of ciCNEs in *C. intestinalis* embryos. (A) Dorsal view of the head of a tailbud embryo electroporated with Pax6_ciCNE2. Reporter expression (arrow, blue stain) is confined to the sensory vesicle. (B) Dorso-lateral view of the head of a tailbud embryo electroporated with Meis_ciCNE10. Reporter expression (arrow) is localised to the sensory vesicle, adjacent to the otolith pigment cell (ot). (C, D) Lateral and dorsal views respectively of tailbud embryos electroporated with Meis_ciCNE1. In (C) reporter expression (arrow) is localised to the sensory vesicle, adjacent to the otolith pigment cell. In (D) reporter expression (arrow) is confined to the lateral tail epidermis of the right hand side.

At the tailbud stage *Ciona Pax6* is expressed in the central nervous system, including both the brain and spinal cord [27]. Pax6_ciCNE2 drove reporter expression into a subset of this domain in the ventral sensory vesicle, a part of the brain (Figure 8A). Both Meis_ciCNE1 and Meis_ciCNE10 drove expression into the ventral sensory vesicle (Figure 8B, C) and anterior tail epidermis (Figure 8D), in a pattern similar to the endogenous expression pattern of the *Ciona* Meis gene [15], [17]. All three transgenes also drove expression into the endoderm located to the posterior ventral part of the trunk. This is a common ectopic site of expression observed with the pCES vector, reflecting the expression of the gene from which the minimal promoter is derived. The remaining ciCNEs had little or no activity in tail bud stage *Ciona* embryos: only Nkx2.2/2.4_ciCNE2 showed reproducible expression, with this in the posterior ventral trunk endoderm as described above (data not shown). These cells are distinct from the cells to which the mRNA for this gene localises [15].

These results show that the Pax6 and Meis elements, which drive transgene expression in zebrafish, are capable of driving reporter expression in *Ciona* in a pattern reflecting the endogenous mRNA domain. Although Nkx2.2/2.4_ciCNE2 was able to increase the residual activity of the basal promoter, it did not drive expression in a pattern related to the expression of *Ciona* Nkx2.2/2.4. Other ciCNEs failed to activate expression. These may be CNEs associated with gene expression at other points in the life cycle, and hence not active in tailbud stage embryos.

## Discussion

We have identified a genome-wide set of non-coding elements that are conserved between two representatives of the urochordates, *C. intestinalis* and *C. savignyi*. Due to the rapid rate of neutral evolution of their genomes, these two species are ideal candidates for the identification of functionally constrained sequences, and have enabled the generation of a valuable data set for comparison with vertebrate CNEs. ciCNEs are slightly shorter and less well conserved on average than vertebrate CNEs, despite the divergence between the *Ciona* genomes being somewhat less than that between fish and mammals [12]. This suggests, given that these regions are in general candidate *cis-*regulatory elements, that the complexity of *cis-*regulation (as measured by the numbers of transcription factors that bind combinatorially to an element at any one time) might be greater in vertebrates than urochordates. This in turn may reflect the increased number of paralogous transcription factors in vertebrate genomes generated through gene/genome duplications and a greater number of tissue types. A further indication of increased vertebrate complexity, at least associated with developmental regulation, is the larger numbers of CNEs that cluster around individual gene loci; for example there are 10 ciCNEs, compared with a total of 52 vertebrate CNEs, associated with the Meis genes (Figure 1).

To try to further understand the relationship between ciCNEs and vertebrate CNEs from a functional perspective, we assayed a set of *C. intestinalis* CNEs located adjacent to genes that have orthologues in vertebrates that also harbour CNEs. We first adopted a co-injection strategy that has been used previously to characterise vertebrate CNEs in zebrafish embryos, using a minimal beta globin promoter fused to the GFP gene. We then re-assayed all 21 ciCNEs using the well-established *Tol2* transgenesis approach, using a vector containing a *cfos* promoter, again fused to GFP. Although the co-injection strategy resulted in highly mosaic and consequently rather weak GFP expression, we found the same four elements to be active using the *Tol2* approach plus another four ciCNEs that drive weaker expression in a small number of embryos. Thus, we believe the results obtained, at least for the four ciCNEs positive in both assays, are robust and reliable and independent of promoter used. Notably we routinely obtained some non-specific ‘ectopic’ muscle expression using the *Tol2* vector, but this has been previously documented [28].

The positive ciCNE from the *pax6* locus (Pax6_ciCNE2) drives expression in sensory neurons in the spinal cord and cranial ganglia in zebrafish embryos at 48 hpf. GFP expression extends caudally as far as sensory neurons innervating the tail. In zebrafish, *pax6* (represented by two genes, *pax6a* and *pax6b*) is expressed in sensory placodes, the eye and throughout the CNS during neurogenesis although not specifically in cranial ganglia [29]. Furthermore, whilst *pax6* expression is strongest in the ventral spinal cord, sensory neurons tend to originate more dorsally [30]. Similarly, in *Ciona*, *Pax6* is expressed throughout the CNS at early tailbud stage [27], [31]. When the Pax6_ciCNE2 was electroporated in *Ciona* embryos, expression was observed in the ventral sensory vesicle, the most anterior portion of the *Ciona* CNS, and related to the vertebrate forebrain. Thus Pax6_ciCNE2 drives reporter expression consistent with the endogenous pattern of expression of the *Ciona Pax6* gene, and in a pattern that partially overlaps pax6a expression in zebrafish embryos. However, the same element drives somewhat different patterns of reporter gene expression in the two different organisms. Pax6_ciCNE2 is a relatively large sequence (413 nt) and efforts to dissect it were largely unproductive, although a core region encompassing nt 88–244 is able to drive the same pattern of expression as the whole element but in a smaller proportion of injected embryos, suggesting that this core region is either less stable or a weaker enhancer. Interestingly, Pax6_ciCNE2 has been identified in *Ciona* previously but was only tested as part of a larger fragment that encompasses the entire intron 4 region in *C. intestinalis* and as such it does not possess enhancer activity [31].

The *Ciona Meis* gene has 10 CNEs, and two of these exhibit strong and specific enhancer activity in zebrafish embryos. Although the expression patterns driven by Meis_ciCNE1 and Meis_ciCNE10 in zebrafish embryos are very different, both sequences activate expression in neuronal cells. Meis_ciCNE1 in particular activates reporter gene expression in at least four different classes of interneurons and two classes of motor neurons throughout the CNS and is by some margin the strongest enhancer in either assay. Meis genes act as Hox/Pbx co-factors [reviewed in 32] and whilst particularly associated with hindbrain development in vertebrates [33], zebrafish meis genes are expressed throughout the brain and spinal cord as well as in the developing eye [34]. *Ciona Meis* is expressed in the ventral sensory vesicle and the anterior epidermis of the tail and posterior trunk at the tailbud stage [15], [35] and the two Meis ciCNEs direct patterns of reporter gene expression consistent with this pattern.

Dissection of Meis_ciCNE1 resulted in the identification of a large core region (nt 97–384 out of 457) of 288 nt that is sufficient to activate the same pattern of reporter gene expression as the whole element, despite a smaller core region comprising nt 156–310 having no enhancer activity. Similar to Pax6_ciCNE2, the larger core region appears to be a weak enhancer, driving expression in less than half the number of embryos than when the whole element is injected. Both the whole element and core region (nt 97–384) are highly active throughout the spinal cord and hindbrain, consistent with a prominent role for Meis genes in hindbrain development, although the core region activates a smaller percentage of injected embryos. Note that there is very limited reporter expression more rostrally in the mid- or forebrain. Contrastingly, the 3′ region of Meis_ciCNE1 (nt 311–457) is able to activate reporter expression more rostrally in the mid-to-forebrain region of the embryo yet not in the hindbrain or spinal cord. A construct combining the middle and 3′ regions of the ciCNE (nt 156–457) however, results in loss of the rostral expression and restoration of primarily the hindbrain, but also the spinal cord expression patterns. Thus it would appear that this ciCNE has the potential to drive expression in the fore- and midbrain encoded in the 3′ region but that this is repressed by upstream sequences in the ciCNE.

Meis_ciCNE10 is already a comparatively small element at just 108 nt in length and as a consequence was initially dissected into two overlapping regions of approximately 70 nt. Firstly, it was apparent that a majority of the activation potential of this ciCNE was located in the 3′ region. Attention focused on a small core region where loss of different motifs resulted in loss of enhancer activity. Strikingly, a minimal region of just 24 nucleotides (nt 71–94) is able to drive reporter expression in the same pattern as the full length element. However this minimal region was no longer able to activate expression in *Ciona* tailbud embryos. This suggests that mechanisms of activation are subtly different between *Ciona* and zebrafish.

Hhex_ciCNE1 is located in the single intron of the *Ciona Hex* gene. This ciCNE drives reporter expression very specifically (in both assays) in a small population of cells located either in the yolk sac or in the circulatory system, with a morphology reminiscent of monocytes or macrophages. This would reflect a role for Hhex_ciCNE1 consistent with that of zebrafish *hhex* in early haematopoeisis [20], [36].

We also note that the three ciCNEs that tested positive in *Ciona* tailbud embryos also showed the strongest phenotypes in zebrafish embryos, while the ciCNEs that were negative in *Ciona* tailbud embryos had limited or no impact in zebrafish. Whether this apparent association is meaningful is difficult to determine, as *Ciona* transgenesis only assesses construct activity up to a specific point in the life cycle, the tailbud stage. However this stage does present the canonical chordate bodyplan and active neuronal differentiation. One possibility is that *Ciona* enhancers active at this time point are more likely to also be active cross-species, for example reflecting constraint on underlying regulatory circuitry imposed by the use of similar suites of transcription factors to establish the conserved chordate body plan in the two lineages. In this respect we note that one of the few previous studies to demonstrate cross-species enhancer activity between *Ciona* and vertebrates also found tailbud stage enhancers to be active in vertebrates, in this case for two enhancers associated with the *Ciona Hox1* gene [37]. However, we cannot unequivocally conclude this without exhaustive testing for activity amongst the other ciCNEs at other life cycle stages in both *Ciona* and zebrafish, and as such it must remain speculative.

In summary, these results demonstrate that at least some of the regulatory logic encoded in ciCNEs can be recognised and deciphered in a vertebrate embryo, directing specific and reproducible patterns of expression in distinct populations of cells during early development. This is in agreement with other studies that have shown that developmental enhancers can function in heterologous contexts in different species (e.g. [38]). However, as we would predict if there has been extensive CRM remodeling, the patterns of expression activated by ciCNEs in zebrafish embryos are not wholly consistent with the endogenous expression of their associated gene, and can in at least one case be driven by a very small sub-region within a ciCNE. Furthermore, it has been established that trans-regulatory changes (i.e. the ability of one species to interpret the cis-regulatory code from another species) also play a role in the reproducibility of enhancer activity [39]. Our data are supported by another recent study that assayed three putative *Ciona* regulatory elements in zebrafish embryos [10], and suggests that the CRM architecture of vertebrate and urochordate CNEs is very different. We speculate that control is mediated by regulatory cross-talk via a limited number of transcription factors, rather than accurate deciphering of whole ciCNEs as CRMs.

In the vertebrate lineage it is now well established that the most highly conserved regulatory elements are associated with developmental transcription factors, remaining largely unchanged at least since the divergence of cartilaginous fish around 500 million years ago (MYA). However, with just a few exceptions [8], [9], vertebrate CNEs do not show strong sequence similarity to non-vertebrate sequences. In this paper we have tried to examine the reasons for this paradox. Recently, a comparison of vertebrate and *Ciona* conserved non-coding sequences identified between 150 and 200 short stretches of conservation, termed oCNEs (av. 45 bp at 55% identity) [10]. Surprisingly, oCNEs are not found in syntenic locations in vertebrates and urochordates, but are located close to different developmental regulator genes, suggesting they have been co-opted into novel CRMs and regulatory networks, possibly as a result of major re-arrangement events. 65 oCNEs are embedded in our ciCNE set (we would expect no overlap by chance), in agreement with our hypothesis that CRMs have been extensively re-modeled, and that even the small minority of shared sequence ancestry has been re-deployed into new regulatory elements and networks. Indeed, these two complementary datasets hint at the extent of re-structuring of gene regulatory networks early in chordate history, and contribute to our understanding of the processes of evolution within gene regulatory networks in different lineages. A second important contributing factor to CRM re-modeling might be the result of the continued and rapid evolution of ancestral chordate CNE sequences in the urochordate lineage but many more urochordate genome sequences are necessary to measure this. Finally, the location and spatial organisation of multiple CNEs around genes, such as at the meis loci, also show no obvious relationship between lineages. A majority of CNEs are downstream of vertebrate meis1 and meis2 genes or in 3′ introns, whereas the *Ciona* meis gene has no downstream CNEs and all are either upstream or in 5′ introns. If vertebrate and urochordate CNEs have evolved from the same ancestral sequences then there must have been a great deal of local rearrangement of these sequences in early urochordate evolution (vertebrate CNEs remain co-linear across all species and the ciCNEs are co-linear between *C. intestinalis* and *C. savignyi*). Given these observations, we conclude that urochordate and vertebrate CNEs emerged and evolved largely independently.

Conservation of CRM function in the absence of sequence conservation or ancestry is not surprising. There are many well-documented examples of regulatory conservation with low or no sequence conservation [40], [41]. Because transcription factor binding sites are small and degenerate, they can easily arise by chance within a short stretch of genomic sequence thereby making existing binding sites redundant [42], [43]. In this way, previously established regulatory regions can become highly divergent, or new sequence regions may be recruited as regulatory sites, without an overall change in function. Alternatively, extensive re-wiring of the regulatory code can create a new set of CRMs that still co-operate within the GRN to achieve the same output. This is supported by the fact that divergent expression profiles of orthologous sets of zebrafish and *Ciona* genes can still result in similar body plans [44].

Despite an apparent lack of direct sequence ancestry, CNEs from vertebrate and urochordate genomes will not have evolved completely independently. They are associated with the same genes and regulatory networks. Consequently, as we demonstrate here, a number of ciCNEs tested are recognised, at least in part, by specific developmental regulatory states (i.e. a set of transcription factors) when injected into the genome of a species that has been evolving independently for over 500 million years. In essence, this reveals that in several cases vertebrate and urochordate CNEs represent different solutions to the same problem, ensuring that similar cohorts of transcription factors active in a particular cell type switch on the same target gene.

## Methods

### *Ciona* CNE identification

The *C. intestinalis* repeat-masked genome (version v2.0) was retrieved from the Joint Genome Institute website (http://genome.jgi-psf.org/Cioin2/Cioin2.info.html). The *C. savignyi* repeat-masked genome (version v2.1) was retrieved from the Sidow lab website (http://mendel.stanford.edu/sidowlab/Ciona.html) at Stanford University Medical Centre [45]. For the BLAST similarity search, the *C. savignyi* scaffolds were split into 500 kb fragments overlapping by 200 bp using the EMBOSS [46] program splitter. The *C. savignyi* fragments were then searched for similarity against the sequence of the *C. intestinalis* genome using MegaBLAST [47]. MegaBLAST was run with word seed length 20 bp, mismatch penalty -2 and e-value threshold 0.001, as described previously for the Fugu-human whole genome comparison [5]. This search returned 177,708 matches between the *C. savignyi* sequence fragments and the *C. intestinalis* genome. In line with Fugu∶human comparisons [5], only alignments at least 100 bp long were retained, thus reducing the set to 73,728 sequence hits.

### Annotation of ciCNEs

The *C. intestinalis* conserved sequence elements were first screened for evidence of transcription according to Ensembl *C. intestinalis* annotation (release v40) using Ensembl Perl API [23]. Elements overlapping exons or containing in total more than 10 bp of repeats were removed. Conserved elements were further filtered by searching for similarity against the EMBL EST, Rfam and the microRNA registry using MegaBLAST (e-value cut-off 0.001). All elements with matches to the non-coding RNA databases were removed and elements with more than three matches to expressed transcripts from EMBL were also removed. In addition, because analysis of duplicated elements was beyond the scope of this manuscript, *C. intestinalis* elements matching multiple locations in *C. savignyi* were removed, too. The final set consists of 2,336 *C. intestinalis* CNEs (ciCNEs), where for 1,817 ciCNEs there is no evidence of transcription and for 519 there is little evidence of transcription (up to 3 matches to expressed transcripts from EMBL)

### Identification of *Ciona* CNE-associated genes

The nearest protein-coding genes (i.e. genes with the nearest TSS) to ciCNEs were retrieved using Ensembl Perl API. 190 of the 2,336 cCNEs were in sequence fragments that did not contain any genes. The remaining 2,146 ciCNEs were assigned to 1,289 protein-coding genes. The human orthologs of the ciCNE-associated genes were retrieved using Ensembl Perl API accessing the Ensembl Compara database, *C. intestinalis* Ensembl Core and *H. sapiens* Ensembl core database (Ensembl release v43).

### Protein domain enrichment analysis

This was performed as previously described for nematode CNEs [7]. In brief, we downloaded InterPro domains of all human and ciona genes from Ensembl [22]. Using a custom Perl script we converted all domains to their top-level parent domain based on InterPro annotation hierarchy [48]. We removed domains present in fewer than 10 genes. We calculated the enrichment of each domain in CNE-associated genes versus the rest using the log-odds ratio test in R and accounted for multiple testing using the Benjamini and Hochberg method [49].

### Zebrafish embryo injection

CNEs were amplified from Ciona genomic DNA by PCR and assayed in zebrafish using the *Tol2* system [50]. The PCR products were cloned into the pCR8/GW/TOPO vector (Invitrogen) and then into a *Tol2*GFP construct [51], using the Gateway LR Clonase II enzyme (Invitrogen). Transient transgenic zebrafish embryos were screened for GFP expression at 24 hpf and 48 hpf.

### Mutagenesis of Meis_ciCNE10

Mutations in the 24 nt sequence of Meis_ciCNE10 were generated by mutating the wild type sequence already inserted into the tol2 vector using the ‘QuickChange II Site-Directed Mutagenesis Kit’ (Agilent Technologies).

### *Ciona* electroporation

Putative ciCNE fragments were directionally cloned in 5′ to 3′ orientation into the β-galactosidase based reporter vector pCES, which uses a minimal promoter derived from the *C. intestinalis FoxAa* gene [52]. Adult *C. intestinalis* type B were collected from marinas on Hayling Island, South England, and maintained in a re-circulating sea water aquarium at 12°C. Gametes were removed separately by dissection, eggs fertilised in vitro and the chorion removed chemically [53] within 15 mins of fertilisation. Electroporation of fertilised eggs was carried out as described, [54], with modifications [55], using 40 µg of construct DNA. Embryos were cultured until the tail bud stage before fixation in 0.2% glutaraldehyde for 30 minutes in sea water, two washes in PBS and transfer to staining buffer (3 mM K_5_Fe(CN)_6_, 3 mM K_3_Fe(CN_6_), 1 mM MgCl_2_). They were stained in staining buffer containing 4 mg ml^−1^ Xgal at 37°C for 12 to 72 hours. All experiments included a negative control (the pCES vector without an enhancer inserted) and a positive control (the *Ciona βγ-crystallin* enhancer [55] in pCES). All negative controls showed no reporter expression, and positive controls showed at least 50% of embryos with palp and/or sensory vesicle expression, reflecting a typical rate of successful electroporation by this method [56].

## Supporting Information

Dataset S1 Co-ordinates of ciCNEs in C. intestinalis (version 2.0).
(XLSX)

Figure S1 Boxplots of the length distributions of the 2,336 ciCNEs (cCNEs) described in this manuscript and the 1373 human CNEs (hCNEs) from [5].
(PDF)

Table S1 Genes associated with CNEs in both human and C.intestinalis (including human orthologues).
(DOCX)

Text S1 DNA sequences of ciCNEs injected into zebrafish embryos.
(DOC)
