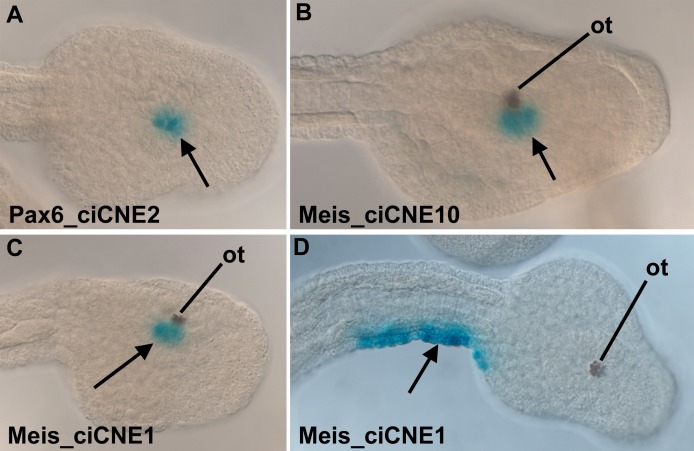# Correction: Parallel Evolution of Chordate *Cis-*Regulatory Code for Development

**DOI:** 10.1371/annotation/1fc82a4b-f9ea-461a-8987-1152078688e4

**Published:** 2013-11-28

**Authors:** Laura Doglio, Debbie K. Goode, Maria C. Pelleri, Stefan Pauls, Flavia Frabetti, Sebastian M. Shimeld, Tanya Vavouri, Greg Elgar

Panels B, C, and D in Figure 8 were labelled incorrectly. For the correct figure, please see 

**Figure:**