# Supplementary figures and images for: Parallel Evolution of Chordate Cis-Regulatory Code for Development
Source: PLoS Genet. 2013 Nov 21;9(11):e1003904. doi: 10.1371/journal.pgen.1003904 (PMC3836708; doi:10.1371/journal.pgen.1003904)

Supplementary Figure 1

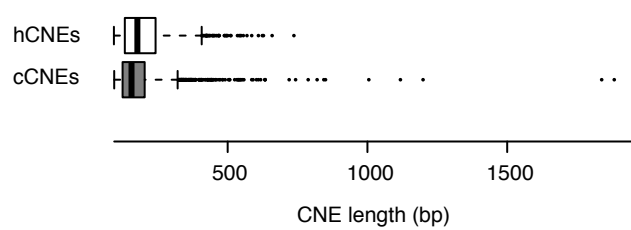

Supplement: Figure S1 — Boxplots of the length distributions of the 2,336 ciCNEs (cCNEs) described in this manuscript and the 1373 human CNEs (hCNEs) from [5]. (PDF) [file pgen.1003904.s002.pdf]
