# Supplementary material for: Parallel Evolution of Chordate Cis-Regulatory Code for Development
Source: PLoS Genet. 2013 Nov 21;9(11):e1003904. doi: 10.1371/journal.pgen.1003904 (PMC3836708; doi:10.1371/journal.pgen.1003904)
Supplement: Table S1 — Genes associated with CNEs in both human and C.intestinalis (including human orthologues). (DOCX) [file pgen.1003904.s003.docx]

Table S1. Genes associated with CNEs in both human and C.intestinalis (including human orthologues)

Ciona-CNE gene Ciona cne coordinates (JGIv2) Ensembl homology type Human-CNE gene Human Gene Name Human CNE coordinates (NCBI35)

ENSCING00000000267 scaffold_1778 6020 6190 ortholog_many2many ENSG00000197327 7 156536733 156536976

ENSCING00000000732 scaffold_784 25811 25947 ortholog_one2many ENSG00000196431 CRYBA4 22 25461182 25461294

ENSCING00000001270 scaffold_88 210497 210605 ortholog_one2one ENSG00000152804 HHEX 10 94440504 94440720

ENSCING00000001700 12q 469798 469950 ortholog_one2one ENSG00000186153 WWOX 16 76945337 76945434

ENSCING00000002253 12q 2962678 2962960 ortholog_one2many ENSG00000020633 RUNX3 1 25139281 25139440

ENSCING00000002422 12q 2803062 2803186 ortholog_one2many ENSG00000111432 FZD10 12 129169060 129169268

ENSCING00000003209 scaffold_186 69301 69409 ortholog_one2many ENSG00000108001 EBF3 10 131567918 131568120

ENSCING00000003763 12q 2281054 2281174 ortholog_one2many ENSG00000168772 CXXC4 4 105703876 105704129

ENSCING00000004086 14q 2085278 2085515 ortholog_one2many ENSG00000179315 15 33333672 33333870

ENSCING00000004120 14q 2219147 2219374 ortholog_one2one ENSG00000171540 OTP 5 76975949 76976062

ENSCING00000004285 4q 1561747 1561984 ortholog_many2many ENSG00000185274 WBSCR17 7 69356106 69356348

ENSCING00000004922 2q 2586197 2586372 ortholog_many2many ENSG00000151615 POU4F2 4 147931917 147932119

ENSCING00000004922 2q 2586197 2586372 ortholog_many2many ENSG00000152192 POU4F1 13 77874883 77874985

ENSCING00000005285 scaffold_63 225812 225916 ortholog_one2many ENSG00000164093 PITX2 4 111913145 111913320

ENSCING00000005356 10q 2049350 2049786 ortholog_one2many ENSG00000134138 MEIS2 15 35114843 35115005

ENSCING00000005356 10q 2049350 2049786 ortholog_one2many ENSG00000143995 MEIS1 2 66573839 66573948

ENSCING00000005904 10p 1045804 1046014 ortholog_one2many ENSG00000009709 PAX7(pax3, no CNEs) 1 18782436 18782554

ENSCING00000006167 1q 972592 972702 ortholog_one2many ENSG00000157985 CENTG2 2 236649282 236649380

ENSCING00000006231 10q 3237765 3238060 ortholog_one2many ENSG00000125816 NKX2-4 20 21310986 21311351

ENSCING00000006503 9q 3321856 3321958 apparent_ortholog_one2one ENSG00000007372 PAX6 11 31782142 31782539

ENSCING00000006930 1q 6452341 6452629 ortholog_one2many ENSG00000169554 ZFHX1B 2 144979311 144979453

ENSCING00000006974 9p 355687 355856 ortholog_one2many ENSG00000123636 BAZ2B 2 160078962 160079157

ENSCING00000007099 7q 4214050 4214418 ortholog_one2many ENSG00000006377 DLX6 7 96278318 96278559

ENSCING00000007099 7q 4214050 4214418 ortholog_one2many ENSG00000144355 DLX1 2 172756947 172757095

ENSCING00000007252 14q 1960018 1960130 ortholog_one2one ENSG00000109133 TMEM33 4 41700539 41700664

ENSCING00000008254 10q 529081 529330 ortholog_one2many ENSG00000169946 ZFPM2 8 106171585 106171751

ENSCING00000008859 4q 4291116 4291337 apparent_ortholog_one2one ENSG00000165588 OTX2 14 56337559 56337679

ENSCING00000009022 3q 4982436 4982766 ortholog_one2many ENSG00000137834 SMAD6 15 64781543 64781675

ENSCING00000009163 5q 5280603 5280789 ortholog_one2many ENSG00000185920 PTCH1 9 95285522 95285646

ENSCING00000009523 3q 1074597 1074751 ortholog_one2many ENSG00000140262 TCF12 15 55212982 55213112

ENSCING00000009523 3q 1074597 1074751 ortholog_one2many ENSG00000196628 TCF4 18 51140501 51140602

ENSCING00000009645 5q 870742 870906 ortholog_one2one ENSG00000164853 (PHOX2B like) 7 1041417 1041757

ENSCING00000010365 3q 5066488 5066632 ortholog_one2many ENSG00000103494 16 52256507 52256653

ENSCING00000012645 3q 2930972 2931136 ortholog_one2many ENSG00000121297 TSHZ3 19 36462723 36462970

ENSCING00000012645 3q 2930972 2931136 ortholog_one2many ENSG00000179981 TSHZ1 18 71051665 71051769

ENSCING00000012645 3q 2930972 2931136 ortholog_one2many ENSG00000182463 TSHZ2 20 50813585 50813733

ENSCING00000012916 1q 156931 157049 apparent_ortholog_one2one ENSG00000004848 ARX X 24706318 24706436

ENSCING00000013048 9q 491093 491271 ortholog_one2many ENSG00000136944 LMX1B 9 126314594 126314702
