# Supplementary material for: Parallel Evolution of Chordate Cis-Regulatory Code for Development
Source: PLoS Genet. 2013 Nov 21;9(11):e1003904. doi: 10.1371/journal.pgen.1003904 (PMC3836708; doi:10.1371/journal.pgen.1003904)
Supplement: Text S1 — DNA sequences of ciCNEs injected into zebrafish embryos. (DOC) [file pgen.1003904.s004.doc]

Ciona CNE sequences plus 50bp up and downstream. Bold indicates positions of primers used and hence extent of sequences injected

Pax6_ciCNE1

>JGI2:9q:3321806:3322008

ATCAAACTGGGCCGTTACTTTTCTCTACGGTCCGTCACTTTTTCTCCGG**GAACACTGATCTACACCGAG**CAACAACAATAGCCACCGAACCTTGTTCCCACGATGGCCGAGAAAAGGAAAAAAGCGGGGGGATTC**CCCGATTACGTATATAGTAGCATCG**ACGAAACATTTCTTGGATTTTGGGAGCCGTCGTCATCCAAATT

Pax6_ciCNE2

>JGI2:9q:3328137:3328649

TTGCCTCTCTAAAATAATTACGTTGAGCTTAAACCATCAACG**GGTCTCTGGTTTCCATTAGTTG**AAAAAATAAAAACTATTTCCCGACCACTGACAGCCGTATTAATGATTAATTGGACGGGATTTCAAGACGATTCCCGAGCCGCGTTCGAAAATAAATAATAAAAGCTCTTTGGATCTCTCGCCCCGTCGCGTCACGTAAAGATCCGTCGACCGCAACCGGCCAGCCCAGCCCCGCATTTCCTATCTGACGCTGCAGCTAATTCCAAGATAAACCGAGTTATTATGAATCAAGTTGCACCGCGATCGATACCGGCGTGTCATCGATCCGTCGGCGGGCTCTCGACGACCAAAAACCCCGCCGAAGTTTCGCTTAAAACCCGGCCTACTCGATTATGAGCGATCGATTTTGGAGAGAATGAAAGACATCCGTGACACGG**CCAGAGCTAAGTAGTTTGTTAGC**GAATTCTGTTGACATTCCGTAGCTTGCAACATAACTCTATTTCAAGCCTG

Otx_ciCNE1

>JGI2:4q:4291066:4291387

CATTGAGTGCTGGGCAAAGTTTCCCTGTCGTGTTGAAGAGAAGTGG**TTTGGATTAAACACACTTCAGACG**TGTGAAAATTCGAGTTGGTTTCAAAAACAAAGTGCGAACGGTAGCGCAAAGAGCGTCGTTGTTGTGACACTGCGCTCGTTCCCAAGCCAGCTCGTATAATAGAAAAACATAAACGCGAAACCTCAAGCAGGTACTCCATCAGGTCCATTCACATAAGCAGCTACTTGGACATTTTCGCACCTTCTATAATG**GGCTTGCCAGCGCTTTGTGAT**CGATCGGGGCCAACTGAAATAGGCTCGTTAACCGTGAACG

Nkx_ciCNE1

>JGI2:10q:3237715:3238110

CCTTTTGCTACTGAGGTTTAGTCAAACATACACA**CATACACTCTTGGACGTATGC**ATTAAAATTACCTTTGCCAATGCCAATATAGCTCGCACTAATGGCGACCTAACAAAATTCGCTTCTCATATACCGACTGTAGTGCTTACTATAGCCCTCGTCTCATCTCTTAGGCCATCTTAAAGGGCGCTCTGGGATATGTTGTAAGTGCATTTCGAGTCTAAAGTAGCCTGCCCATCGGGCTCAAGTCCCCCGCTCAACAAGAAGAGCCTCCATAATTCAACATTAATTCATTTTACTAGTGTGTCCATAGCTAAGGGTGCTTACCAGCATTT**CCGTGGTTATGTACACTCGAA**ACGCGGCATGTTGCTTTGCAAACAAGACACTTTCCCCGAATGATA

Nkx_ciCNE2

>10q JGI2:10q:3242400:3242710

ATATCTATCCGATTTCACGTTACAGGTTTGTGAAAATTATTCTT**GCTTTATTTTGCATTAAACCGG**GCAATTGTTACGTATCCGGAGAAACAATCACGACAACCAAAATACACAGAAAGCAAATAAACTCTGACAGGCCGTGTGACTTCCACGCTAGAGTTGTCTTAAGCTCACACCAATCAGTGATATCAAAGAGGGCTCTATGACATCATAATACGGAGTCTGGAACTTGTTGGCATAA**GCGCTTTGTGGCCCAAACTT**TCAGCACCCGAATCCGTATAGCTCACAAAACGGCTATACCATCCAATCAT

Nkx_ciCNE3

>10q JGI2:10q:3244024:3244297

AGTTATTTGGCAACGATAATGAAACGACAAGGTTCCTCTCCTGGAATTA**GGAATTTATTTGTGAATGGATAC**AATCTAGAACTCAGGTAACGGCATTGTCCGTGTTTCTTACGTCACAATGGTCAGATCACCGGCTCAGCGGGGGAAAACCCACGTCACTCAAAAGCACCCCGCTGGGGAAGATTAGTTCACAAATTAAGTCA**CGAACTTTTTCGTTGAACTTG**AACTGTATTGGCACAAGGGAAAACTAAATTCAGAAATGGTAAAAATCGGG

Otp_ciCNE1

>JGI2:14q:2219097:2219424

AGTCATAGCCGGTTAGATACGTCGACGTCGTTTACGGGCAA**ATTTCTGCGATTAATTTTCGTG**AATTTTATTTCCGTACGACGCCTCTGTTTCAAGCTAAACTTTAGTCTTCCATTTGTCTGTTTATATCAAAGCAGTTTGACTTCGATCTTCGACAGGCCATTGACAGTCATACCCAAATCAATACACCCTCGGCGAAGGGAATATTTACACGCGAAATAATACGCAGAAAATTTCATAAATATTTAATTAAATAATT**CAACTTATGACAATAAATATTCT**AAAAAAAGACGATTTTTTTTAACCGTAATTGCTCTAATAAAGAAAT

PhoxB_ciCNE1

>JGI2:5q:870692:870956

AAACGAAATGCCAACGAGCAAAAGAAAACCTATTTCCACGCCTACCGAC**GCAAGAACGACAAATTACCAC**GTTTCAATTATAGTTTCCCCGAGCCAGCGTAAATAATAGCGAGCTTTGGCGAAATCAAATTTTGTCGAGGGCTTACTGAACAAGAGAATAGATCAGAGAAGTGGTGACTTGACAAATCCTAGGCT**GGATTTTCTTCGTATGTTTCAT**CCTTACCAATACCGCACGCAAGCTGCTGGTCGTAATGATAGGCTTTCG

Meis_ciCNE1

>JGI2:10q:2049300:2049836

TCCCATCTCTGCTTATTATTCTTGTATCGGTAACGGCGACCGGTCCCAGG**CCGGTCCAGGGTTGTAAAAC**GTCGACGAGTTCGGCGAGGGGCCGTAAATCACTCGATGCGTTGGTTTGGAGGGAGGAGAGTTACAAGCGCTCTCGGCAGGGTCATCTAAAGGTGAAGCCCGGGTCAGGATCAGGTGGCTCTCCCGAGACGTTCCGGAGGACGCCCCCTCTCAGCGCGCATTGCACGATCCGCTTTCGCTTCATTAAAGTTTATAGCTCGCTTTATTACGGCGCGTTCGCCTACGCCAAAGGCCATCGATTAAACGTCGCGAAGAGGTCGGGGTCTTGATCTTGCTCCCCGGTCCAGTGAGCTCCTTTCCCCGCAGATTAATTGCGAATGGCCCGTGCTCGGCCTCAACAGATGCTGCCGATTAAACTTGATTATTTTTCCGCCCTTAAGCCTAAATTGCGCGAGGATTAA**TTTCGTTCAATCAATTTGTGGC**GATGTCGGCACAGCAGAAGGCGGTGATCCGGGCGTAAGTGATCCG

Meis_ciCNE2

>JGI2:10q:2050261:2050506

ACATTGGTTCTACTGTTTGGGTTCGCTTATCAATCTGGCCTTCGT**GTCGATTTTCGAGTTTACGTCG**AAACTCGAGACTCGTTGCCTCTGCCACGTCATCCGTCGCTAAGCAACCGGAACTGACTGGCCAGGAGGCTTCGCGTTGACCGATCTGTGACATAATAATGAAGATGTATC**GTCCAAGTGAAAGGGGAGCAC**GAAAAATGGGATTCGAATTTCAGAGAGAGTTCGTATAAATTGCAGCCG

Meis_ciCNE3

>JGI2:10q:2052358:2052674

GGCCGAAATTAGCTAGCGCCGCCCAAGGAAATGTCACCGCG**CAGAACTGGCATTTATCGACG**CGCCGGTCGCCATTTTGTACTCGCTGATGGCTGCTGTCAATATGACATCTTGACGTAGACGGAGGCGATTTGTTTGCGTCACTGCGGCTTGGCAAGTAGGTGCAGCGCGCTGGTAGATCATAATTAGCAAGTGGACGCAAAGGCGGCATTGGAAACAATATTCCCAGGCGTGAAAAGCGAAATAT**GATGAGACAAATATGGCCAGC**GTGAAATCGCGCTGTGAGTGCTCTCTCCAGACGTCCAACTTGACGGAAT

Meis_ciCNE4

>JGI2:10q:2054580:2054950

CGCGAGTTTCTGAGATGCTAGAGCGGCTGTAACAATCTATTCTTGCATAA**GCTGTCGTTCACAGACATAAC**TTCATTACCTTTGACGGGTAGGCGCGTTTTAATGGTGCCTTGTCGATATATTCCAAAGCCAGGTCATTAATAACGGAATTTGACGTTAAACGGCGCCGCATATGCCCGAAGTTCTAAACGCTCTGGCAATTATAACTGCACGGAAATTGAATTAGAAATAAATCGAAGTTCGATGATCTGACGGGCGCTCTCAAACTCAAATTCGATTTGGATGAATATTCAATACGCAATCG**CGACCGAGACGGAAATTGAA**CGAAAAAGAATTGCGCGCTTTCCAGACCCGAGACTTTAATACGTGAC

Meis_ciCNE5

>JGI2:10q:2063167:2063455

GGAGGCGAGCAAATTCCACGAGCAAATAGAAAAACAGCCGAGTTTCATCG**GGGTTTGTTTTTGAGGCGAG**CGAGGGCCAGCAAGCACGTTTTATCACGCTTGGCTAGAAAAGAAGAAGCCGTCGTAGTATTGGGCACAGTGACGCATACTCTGGCCTGTGTCAGTGTGATTCGGACAGCAGAGAAAAGGCTTATATGTAGCCTTCTAGTCTTGGCAAC**CTGCAGAGCAGTGAGATTAAAT**GTCGACCTGCGTGCTTTCGTTTCAGATCGTTGATAGGTGCCTAACTCAG

Meis_ciCNE6

>JGI2:10q:2067413:2067819

AAATTAGCCGTCATCGGCGCCGTCAAAGACGTCACGTTGACGTA**TGAATTACGCAGTACAATCACG**AATATTGGTAGGAATTTGGATCACTGGTCGCGGGGCCCGCCTAGGGACTTGTGTATTGTGTGAGGGGCTTTGTAGTGGCGGTGTCTCTGCTTCTAATCCCACCGCGCTCGTACAAAGTGCTGCTTCCCAAGGATAGGGATAAAAGGCGACTGGGCTTCGTGGGAAGGCACGACTCAGATCGATCCTTCGATCGTCGAATCTTGGAAGAAGATAATATACAGAGATACTTTTACATGGCGCACCGAGCAGACGAAAGCGCGCATTATCAGAGAAATATCATTTTA**GAAAGCAGCGAAGGTCGATA**CCGGGAAAATCAATGGATTTGTTGCAGGGCCTTGGAA

Meis_ciCNE7

>JGI2:10q:2073566:2073960

AGTCGTATTACGTCGCATAATTTCACGGCATATACGGCGATAGTTCG**ATGTCTGGGACTGCGATATTG**TTACACATGTTTCAATATTTGCGGAACTCGGCGCTCGGTGATTGTGCAAACTCGGCCGGGCTGCGAGCCGCCCACTCCCGCCTCGATAATCATTAATTGCCCGATGTTGAAAGAGCGGCCGACCCTTGTGATCGTCGTTTCTCGAGTGGGGAGGCTTCGGCGCCTCAGAAGCGAATCTTGAGTGACGTCTGCTTCGAACGACCTACGTCGTAACGATGTTTGTTTTTTCTGATCGTGAAAAATTAAAACAAATATTGAAATGAAA**GTACATAATTATAGTACAGACTG**TTCGAAACGGTATGTTTGGCGGCGTTACAGTTCTAACCC

Meis_ciCNE8

>JGI2:10q:2073947:2074169

TACAGTTCTAACCCTTATCTCCAAAGTCCCGTACGAGTAATCATATGC**TCAAAAGGCCTGGTAAATAGC**TTAAAAATTCCAGTATTTAGGGACACCTTTTGTTTAATATTAATTCTGTTTATGCCACGATCGTCTTGGTGTGGGAAAGCCTAAAATC**GTAATGCTAACAAATCGACTGT**CGGCGTAAGGTCGTTTCTTCAACCGCGCAAGTATAATAATAATT

Meis_ciCNE9

>JGI2:10q:2074920:2075118

CGTCAAATTTAAACTTAACCACGGACGCAGTTTTTCGTGGAAGAA**CCAGGTTTACTAGAGCAGGTG**CGATTGTTTGTCGACAGACTGATTGGCGACCACGAATGGCACGATCGCCGCACTAAGGCATGAGTTGTGTTTATTTTA**CAACACTATAAGATCCAAGATGG**ACGAAACCCGCGTATAAAGAAATCCTATGTAA

Meis_ciCNE10

>JGI2:10q:2078121:2078327

TCTCCCCCCTCCGAGAAATGGGTCGCTCAGCAGCACTCTTATTCTAC**AGAGCGTCTTGTATAGCGTTTG**TAATGGGATAAGCGGCCTTTAATGCGTCATAATGCAAACGCGCGCAGTATGTGATTAATATTTCATAATGCA**CTAATAGAAATGCATCGGGCTG**CTTCAGTGCACCTAACGATACGTGTTCTACGTCATAACTGCTTT

Ebf3_ciCNE1

>JGI2:scaffold_186:69251:69459

GGACCCGGCCCATGGGTCGTCCGGGACGAATCCACCAAGTTAGTGGC**GAATCGTCCGAGTTGTCCAC**TGAGAGAAGTCTCTGTATTGATGTGTCCGTTGAGACACTGGCTTGCTGGTTTTAAACTCCACGACGCGTGGCCTTCTATA**CTCTAGTATTCCTGACTAGGA**CAGAAAGAAAAAAGGCGGCGCATGTCTTGGAGTAAGGTTCT

Zfhx_ciCNE1

>JGI2:1q:6452291:6452679

CAGGTCACGCAAATTTGCATACACAGCGGCGAGTCAGCAAGCCGGACGGT**CAAAGCAGGTCAGTTCGGGTG**AACCTGTCACTACGTCATCATGCGTGACGTCACACCGCGGGTGGTGGACTTTCTCCATTACAGCGCTCGCCTAGATCAGGGCAGGATAAAGCTTAATTGTAATCGTAACAAAGAGGAAACGCTCTCCTTATTCTCTCACTTCTCACTCTGCTCTTTTTTGTTTCTCTTTTATTCCCCCTCGCCGTATGTGACAGTGTGGTGCGAATATTAAGCAAAGGAAGATGAGACTCGACAGCATTCGGTGCTC**GGTCCAGGTATGGTCAGGAAC**TCGAAGGGAAATTGTACAAGATACACAGCAGACAGTACAGCAGTTTCGAA

Hhex_ciCNE1

>JGI2:scaffold_88:210497:210605

TAAACACACAAACTAACTTATATAACTAACCCTTTTTATGTTGGAAGCAG**TGAGTATACTTAAGAGTGGTG**ACAAATAGCATTACACGATTATTACGATAAGAAACATTGTATCTGGCGAATATGAAGTTGACTTAGGAATGAATGGGCGCCAC**CGTTTGTTTGTCGCGTCTGG**GTTAATCTGTTGAACATTCTCAATGGTTGGCGAATATGTTTACTATTTAA
